# OBAYA (obesity and adverse health outcomes in young adults): feasibility of a population-based multiethnic cohort study using electronic medical records

**DOI:** 10.1186/1478-7954-10-15

**Published:** 2012-08-21

**Authors:** Corinna Koebnick, Ning Smith, Karl Huang, Mayra P Martinez, Heather A Clancy, Andrew E Williams, Lawrence H Kushi

**Affiliations:** 1 Department of Research and Evaluation, Kaiser Permanente Southern California, 100 Los Robles, 2nd Floor, Pasadena, CA, 91101, USA; 2 Division of Research, Kaiser Permanente Northern California, Oakland, CA, USA; 3 Kaiser Permanente Center for Health Research, Hawaii, Honolulu, HI, USA

**Keywords:** Young adults, Obesity, Diabetes, Metabolic syndrome, Cancer, Epidemiology, Cohort study

## Abstract

**Background:**

Although obesity is a risk factor for many chronic diseases, we have only limited knowledge of the magnitude of these associations in young adults. A multiethnic cohort of young adults was established to close current knowledge gaps; cohort demographics, cohort retention, and the potential influence of migration bias were investigated.

**Methods:**

For this population-based cross-sectional study, demographics, and measured weight and height were extracted from electronic medical records of 1,929,470 patients aged 20 to 39 years enrolled in two integrated health plans in California from 2007 to 2009.

**Results:**

The cohort included about 84.4% of Kaiser Permanente California members in this age group who had a medical encounter during the study period and represented about 18.2% of the underlying population in the same age group in California. The age distribution of the cohort was relatively comparable to the underlying population in California Census 2010 population, but the proportion of women and ethnic/racial minorities was slightly higher. The three-year retention rate was 68.4%.

**Conclusion:**

These data suggest the feasibility of our study for medium-term follow-up based on sufficient membership retention rates. While nationwide 6% of young adults are extremely obese, we know little to adequately quantify the health burden attributable to obesity, especially extreme obesity, in this age group. This cohort of young adults provides a unique opportunity to investigate associations of obesity-related factors and risk of cancer in a large multiethnic population.

## Introduction

For the first time in two centuries, life expectancy may decline due to the rapidly increasing prevalence of obesity [1]. In 2007 to 2008, 4.2% of young men and 7.6% of young women 20 to 39 years of age were severely obese, defined as having a body mass index (BMI) at or above 40 kg/m^2^[2]. Although obesity is a risk factor for many chronic diseases, including diabetes and diseases of the kidney and liver, we have only limited knowledge of the magnitude of these associations in young adults.

Managed care systems are a unique system to study associations between rare outcomes in young adults due to their large populations and the potential for long passive follow-up periods. However, lack of generalizability due to healthy worker bias (i.e., insured versus uninsured individuals) and the potential loss of subjects in epidemiologic studies using members of managed care systems are of concern because these factors can be a major of source of bias. Study subjects may lose their health insurance coverage or migrate out of the coverage area but also may re-enroll based on their employment status or other financial decisions and life events. If subjects who leave the health plan are systematically different from those who remain in the health plan in terms of exposure and the association with health outcomes, the estimates of association between exposure and outcome may be systematically biased. The potential of bias exists in all epidemiologic studies due to low and selective responses to recruitment attempts, survey fatigue, migration of subjects, and other factors. However, the control of this bias can be addressed through careful study design and interpretation of the data. As part of the study design, the potential existence of such bias has to be acknowledged, appropriate measures to assess such bias have to be made, and potential effects of such bias for direction and magnitude have to be estimated. Therefore, these potential biases are extremely important to understand.

The long-term goal of this large prospective cohort of young adults is to investigate the relationship between weight class, metabolic syndrome, diabetes, and obesity-related cancers and their risk factors. The analyses presented here show the detailed cohort demographics, as well as cohort retention and the potential influence of migration bias.

## Methods

### Study design, setting, and subjects

The present project was initiated to study the consequences of obesity in young adults (OBAYA) including cancer and leverages the resources of the Cancer Research Network (CRN) [3], an ongoing collaborative project with the National Cancer Institute (NCI) that is comprised of research programs and enrollee populations at 14 geographically dispersed health care delivery systems across the US. The current cohort includes members of Kaiser Permanente Northern and Southern California (KPNC and KPSC), which are the two largest sites participating in the CRN, but will be expanded to other CRN sites. Kaiser Permanente (KP) California health plans are integrated health care systems that jointly cover about 6.5 million members. Members received their care in medical offices and hospitals owned by KP throughout the state. Members enroll through their employer or the employer of a family member, individual prepaid plans, or state or federal programs such as Medi-Cal and Medicare. The OBAYA cohort is comprised of young adults, 20 to 39 years of age, who are enrolled in one of the KP health plans in California. The primary inclusion criterion for the study is at least one medical visit with a measurement of weight and height between 2007 and 2009. However, the study is continuously updated for new members joining the cohort who either 1) recently joined the health plan, 2) surpassed the lower age eligibility limit (≥20 years of age), or 3) had at least one valid weight and height measurement when this information was previously missing. The study protocol was reviewed and approved by the Institutional Review Boards (IRB) of KPSC and KPNC.

### Follow-up

The cohort is followed by passive follow-up through linkage with data extracted from the KP electronic health records. Information on occurrence of cancers comes from linkage of cohort members to KPNC and KPSC tumor registries, which are compliant with data requirements of the NCI Surveillance, Epidemiology, and End Research (SEER) Program and the North American Associations of Central Cancer Registries (NAACCR). Additional data come from probabilistic linkage to the National Death Index (NDI) and to the state cancer registry. The maximum follow-up on December 31, 2010 was four years, the minimum being one year.

### Outcome and demographic measures

The study used information that is routinely assessed during most ambulatory and hospitalization visits. This information captures administrative datasets containing membership and benefit information including all medical encounters at Kaiser Permanente facilities, out-of-system claims, laboratory and radiology test results, and dispensed prescription pharmaceuticals. The address information is routinely geocoded to the census block level, providing the ability to link to census-based group-level socioeconomic information. Laboratory data are also available from electronic medical records. Using the cohort members’ unique medical record numbers, incident diseases can be identified from electronic records, internal disease registries such as the cancer registry, and also from the state cancer registry and state death files.

Body weight and height are routinely measured during almost every medical encounter and were extracted from the electronic health records. BMI was calculated as weight (kilograms) divided by the square of the height (meters). Based on a validation study including 15,000 patients with 45,980 medical encounters, the estimated error rate in body weight and height data was <0.4% [4].

### Census information

Population counts for California were retrieved from Census 2010 data for California for individuals who were 20 to 39 years of age (n = 10,657,405, accessed from the American Fact Finder 2, www.factfinder2.census.gov on July 26^th^, 2011).

## Results

**Figure 1:**
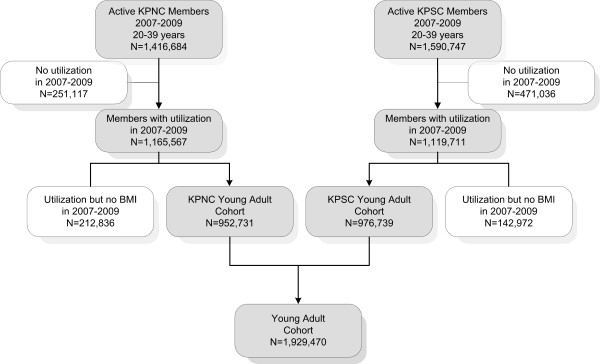
Flow chart of the Kaiser Permanente California young adult cohort.

After exclusion of members who did not have any medical encounters between 2007 and 2009, 2,285,278 young adults were potentially eligible for participation in the cohort study (Figure 1). Of these KP members, 1,929,470 had at least one valid weight and height measurement from electronic clinical records between January 1, 2007 and December 31, 2009 and were included in the study. The cohort included about 84.4% of KP California members in this age group who had a medical encounter during the study period. The cohort showed an almost equal distribution of members across the four age groups (Table 1), a higher number of women than men (57.5% vs. 42.5%), and a large proportion of ethnic/racial minorities with 26.2% non-Hispanic Whites.

**Table 1:** Characteristics of the KP California young adult cohort

**Variable**	**KPNC**	**KPSC**	**Total**
n	952,731	976,739	1,929,470
Sex, male (%)	42.5	42.0	42.2
Age group (%)			
20.0-24.9 y	24.9	26.3	25.5
25.0-29.9 y	24.9	24.4	24.7
30.0-34.9 y	24.5	24.1	24.3
35.0-39.9 y	25.7	25.2	25.5
Race/ethnicity (%)			
Non-Hispanic white	33.8	18.8	26.2
Hispanic	26.9	43.6	35.4
Black	5.7	6.4	6.0
Asian/Pacific Islander	11.7	8.3	10.0
Others	1.8	1.5	1.6
Unknown	20.1	21.4	20.8
Neighborhood education (%)			
Less than high school	18.6	27.7	23.2
High school graduate	20.3	21.0	20.7
Some college or associate degree	31.4	30.1	30.7
Bachelor degree or higher	29.7	21.2	25.4
Neighborhood household income (%)			
<$15,000	10.9	11.9	11.4
$15,000-$34,999	19.1	21.1	20.1
$35,000-$49,999	14.8	15.2	15.0
$50,000-$74,999	21.0	19.6	20.3
$75,000-$99,999	13.9	12.9	13.4
$100,000-$149,999	12.8	12.9	12.8
$150,000 or more	7.5	6.4	7.0
Receivers of Medi-Cal or other subsidized services (%)	1.8	2.8	2.3
BMI class (%)			
<18.5 kg/m^2^	1.8	1.8	1.8
18.5-24.9 kg/m^2^	38.7	34.4	36.5
25.0-29.9 kg/m^2^	31.5	31.8	31.6
30.0-34.9 kg/m^2^	16.1	18.0	17.0
35.0-39.9 kg/m^2^	7.1	8.2	7.7
40.0-49.9 kg/m^2^	4.1	4.9	4.5
50.0-59.9 kg/m^2^	0.6	0.8	0.7
>60 kg/m^2^	0.1	0.1	0.1

The young adult cohort represented about 18.2% of the underlying population in the same age group in California (Table 2). The age distribution of the cohort was relatively comparable to the underlying population in California as evaluated by the Census 2010. However, the young adult cohort has a slightly higher proportion of women and of Hispanics, Blacks, and Asians but a lower proportion of individuals of other races than the underlying Census population.

**Table 2:** Population demographics compared to California Census 2010

**Subject (%)**	**KP California young adult cohort**	**Census 2010**
Total population (n)	1,929,470	10,657,405
Sex, male (%)	42.2	51.1
Age group (%)		
20.0-24.9 y	25.5	26.0
25.0-29.9 y	24.7	25.8
30.0-34.9 y	24.3	24.1
35.0-39.9 y	25.5	24.1
Race/ethnicity (%)*		
Non-Hispanic white	33.1	27.6
Hispanic	44.6	34.5
Black	7.6	4.9
Asian/Pacific Islander	12.5	11.7
Others	2.1	21.4

* For the young adult cohort, information on race and ethnicity are based on administrative records among those with known race/ethnicity.

The current three-year retention rates for young adults who entered the cohort in 2007 is 68.4%, ranging from 58.1% in the youngest adults between 20.0 and 24.9 years of age to 75.6% in young adults between 25.0 and 29.9 years of age (Table 3). To investigate changes in demographic characteristics due to disenrollment, we compared participants who were enrolled into the study in 2007 by their retention status (Table 4). Those participants who were lost to follow-up were more likely to be younger and male but slightly less likely to be from a racial/ethnic minority. Neighborhood education and household incomes were comparable between those retained and those lost to follow-up.

**Table 3:** Retention* of young adults in the health plan

**Year of enrollment into cohort**		**Retention of young adults in health plan (%)**			
		**Baseline**	**1 year**	**2 years**	**3 years**
	**Age (years)**	**%**	**%**	**%**	**%**
**2007**	20.0-24.9	100	87.7	71.5	58.1
	25.0-29.9	100	88.8	75.6	65.6
	30.0-34.9	100	91.3	80.4	71.8
	35.0-39.9	100	92.7	83.4	75.6
	All ages	100	90.3	78.2	68.4
**2008**	20.0-24.9	100	86.7	69.2	
	25.0-29.9	100	86.6	70.6	
	30.0-34.9	100	88.9	75.3	
	35.0-39.9	100	90.4	78.5	
	All ages	100	88.1	73.2	
**2009**	20.0-24.9	100	86.4		
	25.0-29.9	100	84.7		
	30.0-34.9	100	87.0		
	35.0-39.9	100	88.2		
	All ages	100	86.4		
**All years**	20.0-24.9	100	87.0	70.6	58.1
	25.0-29.9	100	87.5	74.0	65.6
	30.0-34.9	100	90.0	78.8	71.8
	35.0-39.9	100	91.5	82.0	75.6
	All ages	100	89.0	76.5	68.4

*Retention is defined as proportion of active members 20-39 years retained in the health plan on December 31 of each year.

**Table 4:** Demographic characteristics of cohort members enrolled in 2007 by three-year retention status

**Variable**	**KP California members with 2007 cohort enrollment**	
	**Retained**	**Lost to follow-up**
n	748,984	308,395
Sex, male (%)	36.2	39.9
Age group (%)		
20.0-24.9 y	18.4	29.2
25.0-29.9 y	23.7	26.5
30.0-34.9 y	27.0	22.8
35.0-39.9 y	30.9	21.5
Race/ethnicity (%)		
Non-Hispanic white	29.9	25.2
Hispanic	35.7	35.6
Black	7.3	5.4
Asian/Pacific Islander	11.3	7.8
Others	2.1	1.5
Unknown	13.7	24.5
Neighborhood education (%)		
Less than high school	23.2	23.5
High school graduate	20.9	20.8
Some college or associate degree	30.9	30.6
Bachelor degree or higher	25.0	25.1
Neighborhood household income (%)		
<$15,000	11.2	11.8
$15,000-$34,999	19.8	20.5
$35,000-$49,999	15.0	15.2
$50,000-$74,999	20.4	20.2
$75,000-$99,999	13.6	13.1
$100,000-$149,999	13.0	12.4
$150,000 or more	7.0	6.8
Receivers of Medi-Cal or other subsidized services (%)	2.6	2.3
BMI class (%)		
<18.5 kg/m^2^	1.5	1.9
18.5-24.9 kg/m^2^	32.2	35.6
25.0-29.9 kg/m^2^	31.9	31.1
30.0-34.9 kg/m^2^	19.0	17.4
35.0-39.9 kg/m^2^	8.9	8.1
40.0-49.9 kg/m^2^	5.5	5.0
50.0-59.9 kg/m^2^	0.9	0.8
>60 kg/m^2^	0.1	0.1

## Discussion

This cohort of young adults provides an unparalleled opportunity to investigate associations of obesity and adverse health outcomes including cancer. Its large population of young adults is relatively representative for the underlying population in California. The availability of clinically assessed height and weight data for nearly 2,000,000 California members of the KP health system provides an outstanding basis for investigating the effects of weight class on health outcomes. In addition, there is substantial racial and ethnic diversity in this cohort, with a large proportion of Hispanics – about one-third of the cohort – and Asians – about 10%. The ability to link to various clinical and administrative databases including high-quality tumor registries, prescription medications, laboratory results, and diagnoses and procedures related to outpatient encounters or hospitalizations facilitates investigation of numerous outcomes of interest.

Health consequences of obesity may vary markedly by sex, race/ethnicity, and socioeconomic status. Whether variations in obesity are a principal reason for disparities in disease occurrence in young adults is unclear. Variations in disease occurrence in young adults have also been attributed to the lack of health insurance in this particular age group, with 40% lacking health insurance [5,6]. Another problem may be underinsurance, which includes electing coverage in plans with relatively low premiums but substantial copayments and high deductibles. A lack of health insurance or underinsurance may result in underutilization of health care services, and consequently, delayed or underdiagnosis of disease. This may bias results from epidemiological studies that do not take insurance status into account and lead to an underestimation of obesity-related risks. Current knowledge gaps of the health risks associated with obesity in young adults include: 1) lack of robust risk estimates for young adults, because most studies investigate adults of all ages without further stratification; 2) limited information on disparities and risk in subpopulations; and 3) lack of information on health outcomes with lower prevalence.

For this ongoing cohort study of young adults, the understanding of potential bias introduced by migration is crucial. The membership in KP, and therefore in the cohort, is dynamic, with individuals continuously joining and leaving the health plan. Retention rates in young adults are generally lower than in other age groups, likely due to the high rate of change in employment and family status in this age group. The current three-year retention rates for young adults who entered the cohort in 2007 is 68.4%, ranging from 58.1% in the youngest adults between 20.0 and 24.9 years of age to 75.6% in young adults between 25.0 and 29.9 years of age. These data suggest the feasibility of our study for medium-term health outcomes with an adequate length of follow-up based on sufficient membership retention rates. Future retention rates cannot be predicted accurately because of ongoing changes in health policy such as the Affordable Care Act; the interpretability of our results is limited to medium-term retention. However, we speculate that the expansion of health care to a larger population as planned in the Affordable Care Act would increase retention rates in this age group known for high rates of uninsured individuals [5,6]. The estimated membership retention is expected to increase slightly by combining KPSC and KPNC as members relocating from one health plan to another will continue to be available for follow-up.

Individuals who were retained in the health plan and those lost to follow-up were remarkably similar with regard to neighborhood income and education. However, those participants who were lost to follow-up were more likely to be younger, male, and non-Hispanic White. They were also less likely to be of unknown race/ethnicity. Differences in race and ethnicity between these two groups can be partially attributed to the introduction of a mandatory assessment of race/ethnicity during outpatient visits for every patient in 2009. Therefore, individuals who left the health plan before 2009 were more likely to be of unknown race/ethnicity. Overall, our data do not suggest major bias by attrition regarding sociodemographic factors. However, factors not considered in this analysis may exist that could introduce bias such as systematic differences between individuals retained and lost to follow-up in health risk factors related to the outcomes of interest that need future consideration.

Although cancer in young adults is one of the leading causes of disease-related deaths in this age group, little attention has been given to risk factors for cancers in young adults 20 to 39 years of age. Obesity [7], diabetes [8-10], and metabolic syndrome [11-13] have all been linked to an increased risk for several cancers, based mostly on cancer incidence in adulthood [9,13-15]. Although young adults in the US have a high prevalence of obesity [16], diabetes [17,18], and metabolic syndrome [19], few epidemiologic studies have focused on these chronic conditions and subsequent cancer risk in this age group. This is particularly important given increasing evidence that a substantial proportion of cancers in young adults likely have a different underlying biology, etiology, and pathogenesis than in older individuals [20,21]. Thus, while studies have reported associations between obesity, diabetes, metabolic syndrome, and many common cancers in older adults, such associations in young adults have yet to be established.

At cohort entry, all members by definition have had health insurance coverage, and this may be perceived as limiting the generalizability of the findings. Indeed, having health insurance is associated with potentially key covariates in disease risk, such as employment status or income. Despite this, there is a substantial variation in socioeconomic status, based on census-based estimates. In addition, uniform health coverage is a substantial advantage as it minimizes the risk of underdiagnosis or delayed diagnosis, which may in turn result in biased risk estimates. This is a potential limitation for cohort studies in which young adults are enrolled without regard to health insurance status, as 40% of young adults in the US do not carry health insurance.

Despite these outstanding strengths, there are some limitations. As a cohort based on electronic health records, the availability of data on covariates of interest will vary, as not all cohort members will take routine advantage of services such as screening for conditions of interest. Thus, there is the possibility that availability of screening data will be linked to disease status. This is mitigated to some extent by the large numbers of cohort members and the ability to define subsets of the cohort by the availability of data or frequency of encounters with the health care system. In addition, the implementation of clinical practice guidelines, such as systematic screening for cardiovascular disease risk factors for all members 20 years of age and older on the first clinical visit and every five years thereafter, will assure broad availability of relevant data without bias as to disease status. On the other hand, particularly for the occurrence of short-term events, the potential for confounding by indication or the prodromal effects of disease resulting in more frequent health encounters will need to be taken into account in the interpretation of findings.

As noted previously, there is disenrollment of individuals from the KP health plan, with overall about 68% of cohort members maintaining their KP health insurance after three years. Retention rates are lowest for the youngest adults and somewhat higher for those in their fourth decade of life. The loss to follow-up is mitigated somewhat for endpoints such as death or cancer due to the possibility of linkage to state and SEER tumor registries or state and national vital statistics registries. The attrition rate does indicate that the ongoing update of exposure or comorbid information may be limited. However, we will be able to explore differences in those who are retained in the cohort as members of KP and those who have disenrolled to determine if there may be systematic biases in important factors associated with exposures of interest, such as body size, or outcomes of interest, such as diabetes, cardiovascular disease, and cancer rates.

Nationwide 6% of young adults are extremely obese; yet we know little to adequately quantify the health burden that can be attributed to obesity, especially extreme obesity, and which population groups are most susceptible to early health consequences. This cohort of young adults and their electronic medical record data provide an unparalleled opportunity to investigate associations of obesity-related factors and risk of cancer and other diseases in a large multiethnic population. These data sources are unusually rich and support the development of nuanced longitudinal care quality indices for preventive and disease management services, such as the Prevention Indices and Disease Management Indices [22,23]. Unlike claims-based quality standards, these indices draw on the full range of clinical and administrative data to define both the population and the delivery of the service over time.

In addition to the aforementioned limitations, the cohort currently has a relatively short follow-up that enables us to draw conclusions on short- and medium term outcomes. Analyses have to be designed carefully to account for systematic differences beyond demographic factors such as body weight and obesity-related conditions to investigate long-term health risks such as cancer risk. Linkage to other databases can help decrease the attrition in this cohort, including internal linkage between Kaiser Permanente regions (restricted to those who stay with Kaiser Permanente), linkage with state death files (restricted to death cases), and linkage with state cancer registries (restricted to those who remain in the state and limited to cancer diagnosis).

Planned future research on the young adult cohort will develop these quality measures in order to identify person-level characteristics associated with both variations in services related to cardiovascular disease, diabetes, and cancer in young obese adults and variations in the care they receive and its consequences for incident morbidity, mortality, and health care utilization. This planned research will also examine differences in the effectiveness of that care in demographically and medically defined subpopulations.

## Abbreviations

BMI: Body Mass Index; CRN: Cancer Research Network; NCI: National Cancer Institute; KPNC and KPSC: Kaiser Permanente Northern and Southern California; KP: Kaiser Permanente; IRB: Institutional Review Board; NCI: National Cancer Institute; SEER: NCI Surveillance Epidemiology, and End Research; NAACCR: North American Associations of Central Cancer Registries; NDI: National Death Index.

## Competing interest

Lawrence H Kushi reports a relevant relationship as Adjunct Professor at the UC Davis Medical School, which is not his primary employment.

## Authors' contributions

Design and conduct of the study: CK, LHK; Collection, management, analysis and interpretation of data: CK, LHK, NS, MPM, KH, HAC, AEW; Preparation of the manuscript: CK, MPM, NS; Critical revision of the manuscript for important intellectual content: LHK, KH, HAC, AEW. All authors read and approved the final manuscript.
